# Is High-Dose Ubiquinone Therapy Before Cardiac Surgery Enough to Reduce the Incidence of Cardiac Surgery-Associated Acute Kidney Injury? A Randomized Controlled Trial

**DOI:** 10.3390/antiox14020243

**Published:** 2025-02-19

**Authors:** Hrvoje Vučemilović, Ruben Kovač, Lada Stanišić, Ana Sanader Vučemilović, Dina Mrčela, Benjamin Benzon, Mladen Carev

**Affiliations:** 1 Department of Anaesthesiology, University Hospital of Split, 21000 Split, Croatia; rkovac@kbsplit.hr (R.K.); mcarev@kbsplit.hr (M.C.); 2 Division of Medical Laboratory Diagnostic, University Hospital of Split, 21000 Split, Croatia; lstanisic@kbsplit.hr; 3 Department of Dermatovenerology, University Hospital of Split, 21000 Split, Croatia; ana.sanader@hotmail.com; 4 Department of Pediatric Diseases, University Hospital of Split, 21000 Split, Croatia; dmrcela@kbsplit.hr; 5 Department of Anatomy, Histology and Embryology, School of Medicine, University of Split, 21000 Split, Croatia; bbenzon@mefst.hr; 6 Department of Anaesthesiology and Intensive Medicine, School of Medicine, University of Split, 21000 Split, Croatia

**Keywords:** ubiquinone, coenzyme Q10, coronary artery bypass surgery, acute kidney injury, acute kidney failure, cardiopulmonary bypass, postoperative hemorrhage, high sensitivity troponin I

## Abstract

Cardiac surgery-related acute kidney injury (CS-AKI) is a decrease in kidney function after open-heart surgery, affecting up to 50% of patients. The pathophysiology of CS-AKI involves ischemia–reperfusion injury, inflammation, and oxidative stress. Ubiquinone is a potent antioxidant, and we hypothesized that it could decrease both the incidence and severity of CS-AKI. The intervention group received ubiquinone (8 mg/kg/day) divided into three daily doses, while the control group received a placebo. The primary outcome was the incidence of CS-AKI, which was manifested as an increase in creatinine ≥26.5 µmol/L or a urine output below 0.5 mL/kg/h for 6 h. Out of 73 patients, 39.7% (N = 29) developed CS-AKI, including 35.3% of the ubiquinone group and 43.6% of the placebo group (*X*^2^(1,N = 73) = 0.4931, *p* = 0.4825). The secondary outcomes revealed that the ubiquinone group experienced reduced postoperative bleeding, with a median (IQR) drainage of 320 mL (230–415) compared to the drainage of 420 mL (242.5–747.5) in the placebo group (t(35.84) = 2.055, *p* = 0.047). The median hs-TnI level in the ubiquinone group was 239.5 ng/mL (113.25–382.75) after surgery compared to a level of 366 (234.5–672.5) ng/mL in the placebo group (*p* = 0.024). In conclusion, there was no significant difference in the incidence of CS-AKI between groups. Postoperative hs-TnI and bleeding were significantly reduced among patients receiving ubiquinone.

## 1. Introduction

Cardiac surgery-associated acute kidney injury (CS-AKI) is a sudden decline in kidney function that occurs after cardiac surgery [1]. The Kidney Disease: Improving Global Outcomes (KDIGO) classification defines it as an increase in serum creatinine occurring within 48 h of surgery [2]. CS-AKI affects up to 50% of individuals undergoing open-heart surgery, and it is the second most common type of acute kidney injury in the ICU [3]. Patients that experience CS-AKI often progress to having chronic kidney disease (CKD) within a few years, with stages 2 and 3 leading to CKD earlier [4].

The complex pathophysiology of CS-AKI encompasses multiple mechanisms with variable degrees of impact, such as hypoperfusion due to low cardiac output in cardiorenal syndrome type 1, hypotension before, during, and after surgery, embolism, inflammation, cardiopulmonary bypass, ischemia with reperfusion, and oxidative damage [5,6,7].

During cardiopulmonary bypass (CPB), a significant inflammatory response causes renal vasoconstriction in response to the conversion from pulsatile to laminar blood flow [8]. Furthermore, hypotension associated with vasoconstriction leads to renal ischemia, depletion of adenosine triphosphate, and death of renal tubule cells [9]. This leads to a reduction in the glomerular filtration rate (GFR) via a tubuloglomerular feedback mechanism and necroinflammation [10]. While the etiology of CS-AKI is multifactorial, ischemia–reperfusion injury (IRI) may be the most significant factor. Mitochondria are crucial in ischemia–reperfusion injury (IRI) as they represent the primary source of free radicals that lead to cellular damage during reperfusion [11]. Diabetes mellitus, arterial hypertension, and tobacco smoking have been associated with oxidative stress, endothelial dysfunction, and diminished oxidative resistance, potentially exacerbating the aforementioned mechanisms [12,13,14].

Patients undergoing cardiopulmonary bypass exhibit a decrease in antioxidant capacity, indicated by postoperative decreases in the plasma concentrations of vitamins C and E and coenzyme Q10 [15,16].

Previous research explored vitamins C and E as protective agents against AKI, with vitamin C showing a reduction of 37% in contrast-induced AKI [17,18].

To our knowledge, there are no studies investigating whether ubiquinone could reduce kidney damage after cardiac surgery. Dare et al. investigated triphenilyphosphonium covalently linked to ubiquinone (MitoQ) in an animal model of prolonged kidney ischemia lasting 45 min. Their findings indicated that MitoQ was successful in preventing kidney damage during prolonged ischemia and subsequent reperfusion [19].

Unlike water-soluble antioxidants, coenzyme Q10 (CoQ10; ubiquinone) is a widely distributed fat-soluble antioxidant found in the hydrophobic regions of cellular membranes. CoQ10 functions as an electron carrier in the mitochondria, where it plays a role in the process of oxidative phosphorylation [20]. Patients with cardiovascular disease often receive HMG-CoA reductase inhibitors, also known as statins, which reduce circulating CoQ10 levels in blood [21]. Low CoQ10 levels could contribute to mitochondrial dysfunction and mitochondrial permeability, potentially triggering cell death [22]. This could potentially aggravate mitochondrial dysfunction already present in patients with heart failure, with mitochondrial dysfunction being one of the triggers of AKI [23]. Tissues with the highest energy requirements, such as the heart, kidneys, liver, and pancreas, are also the tissues with the highest mitochondria and coenzyme Q10 contents [24,25]. Patients with chronic kidney disease [26], diabetes type 2 [27], chronic obstructive pulmonary disease [28], and even periodontal disease [29] exhibit coenzyme Q10 deficiency. Sixty percent of patients with chronic heart failure demonstrated decreased plasma coenzyme Q10 levels, which correlated more significantly with mortality than the NT–proBNP [30]. Ubiquinone supplementation is safe and beneficial, with significant reductions in cardiovascular and all-cause mortality [16].

Considering the abundance of mitochondria in kidneys, low coenzyme Q10 concentrations in patients requiring cardiac surgery and the pathophysiology of CS-AKI ubiquinone supplementation before surgery seemed prudent to evaluate. The upper safe limit of the ubiquinone daily dose is unclear. Depending on the condition, the ubiquinone dose should be from below 1200 to 2400 mg/day [31]. Pharmacokinetic work by Rosenfelt et al. suggests that coenzyme Q10 levels in tissue increase gradually [32]. Therefore, we administered ubiquinone at a dosage of 8 mg/kg/day, divided into three doses post-meal, for a duration of 7 days prior to surgery.

Our hypothesis is that administering high doses of ubiquinone one week before cardiac surgery would provide sufficient protection against ischemia–reperfusion injury during surgery and CPB to reduce the incidence of CS-AKI in the intervention arm of the study.

The primary objective of this study was to explore whether ubiquinone therapy before an elective open-heart surgery decreased the incidence of CS-AKI, as manifested by an increase in creatinine of >26.5 µmol/L up to 72 h after surgery and/or a reduction in diuresis to below 0.5 mL/kg/h. The primary outcome was the difference in the incidence of CS-AKI between the group receiving ubiquinone and the group receiving placebo.

The secondary objectives were to determine whether there were any differences in the stages of AKI according to KDIGO and to examine the effects of ubiquinone on thoracic and mediastinal bleeding in the first 48 h and the difference in myocardial injury detected by high-sensitivity troponin I (hs-TnI). The secondary outcomes were the difference between the incidence of AKI between groups according to KDIGO stages, the blood loss levels in the cardiac ICU 24 and 48 h after surgery, the difference in the levels of high-sensitivity troponin I between groups 24 h after surgery, and the differences in the hemodynamic parameters between groups.

## 2. Materials and Methods

### 2.1. Study Design and Participants

This study was a single center, prospective, randomized, double blind, placebo-controlled trial of ubiquinone therapy for patients undergoing elective cardiac surgery. We enrolled patients from July 2020 to December 2022 at the University Hospital Center Split, Croatia. Patients were admitted to either the Department of Cardiovascular Diseases for additional testing before surgery or to the Division of Cardiac Surgery. The investigators would assess, according to the study protocol, whether the patient fulfilled the inclusion criteria.

Exclusion criteria were urgent/emergent surgery, high-dose vitamin B therapy, warfarin therapy, thrombocytosis (>450 × 10^9^/L), preexisting kidney disease (GFR ≤ 60 mL/min/1.73 m^2^), obstructive uropathy, previous cardiac surgery, liver disease, alcohol abuse, allergy to any ingredient of the ubiquinone capsule, uncontrolled hypertension, and preoperative hypotension requiring inotropes and/or vasopressors. We excluded 19 patients due to preexisting kidney disease (n = 15), previous cardiac surgery (n = 2), liver disease (n = 1), and alcohol abuse (n = 1). Twelve patients decided not to participate.

**Figure 1:**
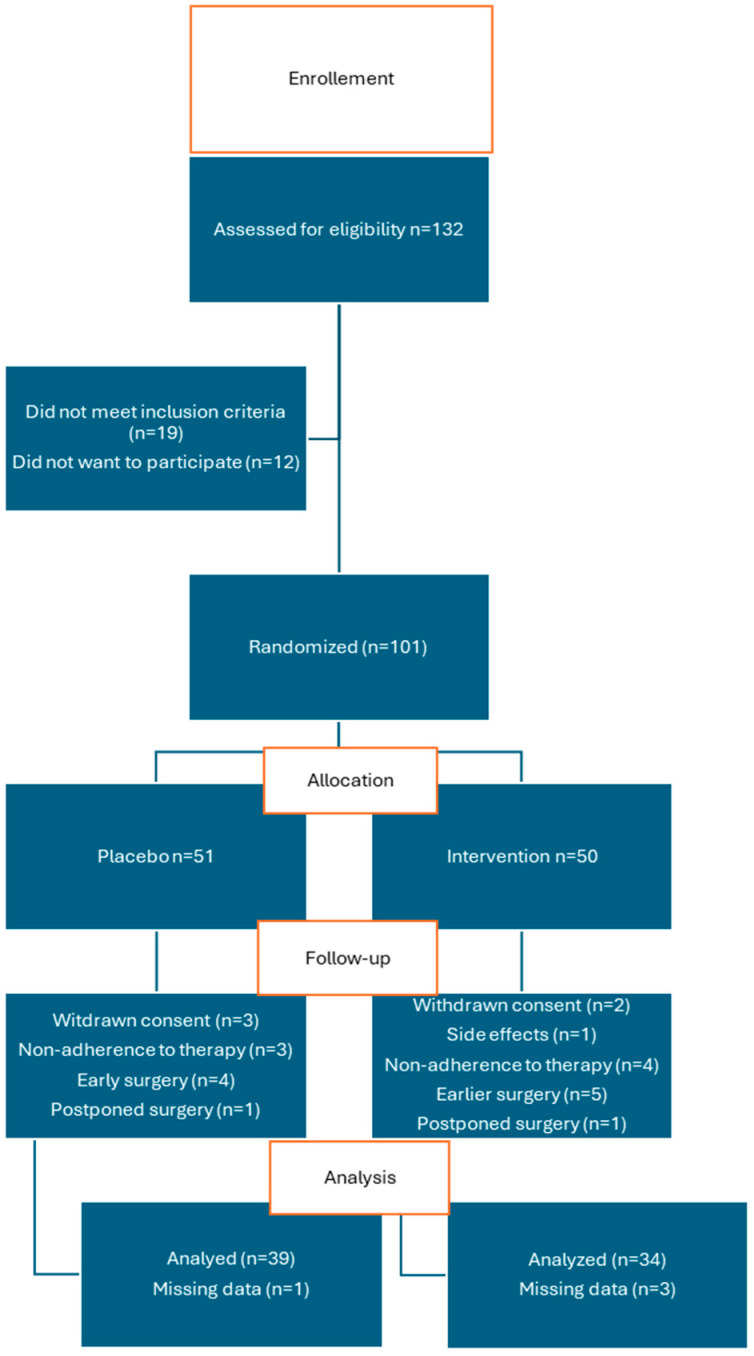
CONSORT flow diagram of the study.

Figure 1 shows the CONSORT flow diagram. We assessed 132 patients for eligibility. We randomized 101 patients into two groups. The ubiquinone group (UQ) received ubiquinone, and the control group received placebo. During the follow-up, nine patients underwent surgery before the completion of seven days of therapy and were therefore removed from the study. We analyzed data from 73 participants.

### 2.2. Ubiquinone Therapy

Patients scheduled for elective cardiovascular surgery were randomly assigned to treatment or placebo group using block randomization. We used an online tool available at www.sealedenvelope.com to create a list of 152 long letters in blocks of 4, 6 or 8 letters. Depending on the allocation, a resident from our department would then choose blisters from box A (placebo) or B (ubiquinone) and deliver them to the patients. Blisters containing placebo or ubiquinone were identical. Ubiquinone was delivered in black capsules, with each capsule containing 100 mg of ubiquinone, while the placebo was provided in identical capsules. The residents were unaware which box contained placebo or ubiquinone. Patients would receive as many blisters needed for one week of therapy. We checked patients every 24 h to determine adherence to therapy and the occurrence of side effects.

Patients received a daily dose of 8 mg of coenzyme Q10 per kilogram of body weight in the form of ubiquinone (Pharma Nord, Vejle, Denmark). The total daily amount of ubiquinone for patients who weighed 65 kg was 520 mg (two capsules in the morning, two capsules after lunch, and one after dinner; 500 mg in total). To compensate for the 20 mg daily deficit of ubiquinone, patients would receive six capsules (600 mg) on the fifth day of therapy. We used the same principle in all patients ensuring that every patient received 8 mg/kg/day of ubiquinone. The duration of the therapy was seven days before surgery.

### 2.3. Ethical Approval

The study was conducted in accordance with the Declaration of Helsinki and was approved by the Ethics Committee of University Hospital Center in Split (ID: 2181-147-01/06/M.S.-19-4). Written informed consent was obtained from all study participants. The study was registered on clinicaltrials.gov under the ID NCT04445779.

### 2.4. Definition of Acute Kidney Injury

Acute kidney injury was defined according to KDIGO guidelines as an increase in serum creatinine by ≥0.3 mg/dL or ≥26.5 µmol/L within 48 h. The definition also included any reduction in urine volume below 0.5 mL/kg/h for 6 h. Table 1 presents more in-depth KDIGO guidelines [2] for staging and defining acute kidney injury and failure, including stages and descriptions.

### 2.5. Coenzyme Q10 Determination

We sampled blood for coenzyme Q10 determination three times: 30 min before surgery in the morning, 30 min after admission to the cardiac ICU, and 24 h after the surgical procedure. Sampled blood was immediately centrifuged and separated. The samples were prepared by using coenzyme Q10 reagens (RECIPE Chemicals and Instruments GmbH, Munchen, Germany), where a reduced form of coenzyme Q10 was transformed into the oxidized form. An aliquot of lipid extract was injected and fractionated by high-performance liquid chromatography (HPLC). The sample components were chromatographically separated and detected by the UV detector. The calculation of the measurements was performed by using the internal standard method via peak areas.

We measured coenzyme Q10 levels in plasma with ClinRep HPLC Kit (RECIPE Chemicals and Instruments GmbH, Munchen, Germany). The samples were prepared and were injected into the HPLC system. The sample components were chromatographically separated and detected by the UV detector (UV/VIS SPD-20AC, Shimadzu Co., Kyoto, Japan).

For sample preparation, a defined amount of precipitation reagent was put into a sample preparation vial. An aliquot of the sample and oxidation reagent was added. The oxidation reagent reduced form of coenzyme (UQH2) was transformed into its oxidized form UQ. The sample was mixed shortly, and an internal standard was added. After this, the sample was mixed vigorously to ensure a quantitative precipitation of the sample matrix. After centrifugation, the sample showed a clear supernatant that consisted of two liquid phases. An aliquot of the upper phase was used for HPLC analysis. The calculation of the measurements was performed using the internal standard method via peak areas.

### 2.6. Patient Preparation and Premedication

All preoperative medications continued until the day of surgery, except for diuretics and angiotensin-converting enzyme inhibitors. Oral anticoagulants were stopped according to the current EACTS guidelines [33].

Patients received premedication with oral diazepam (5 mg) the night before surgery and with intramuscular morphine (0.1–0.15 mg/kg) 30–45 min before arrival in the operating theater.

### 2.7. Anesthesia Induction

After placing standard anesthesia monitoring, as recommended by the American Society of Anesthesiologists, placing two large bore intravenous cannulas, and performing invasive arterial blood pressure monitoring, the cardiac anesthesiologist induced anesthesia using midazolam (0.1–0.15 mg/kg), fentanyl (5–7 mcg/kg), and vecuronium (0.15 mg/kg). The anesthesiologist maintained anesthesia with boluses of midazolam (5–10 mg iv), fentanyl (250–500 mcg), sevoflurane (1–1.5 MAC), and vecuronium. Etomidate was used for intravenous induction of general anesthesia. The anesthesiologist re-administered vecuronium every 40–50 min. The last dose of vecuronium was administered just before the start of sternum closure with sternal wires.

### 2.8. Intraoperative Procedure

All patients were mechanically ventilated in continuous mandatory volume ventilation mode, and the tidal/minute volume, fraction of inspired oxygen (FiO_2_), end-tidal carbon dioxide, as well as inspired/end-tidal sevoflurane concentration were monitored.

Intraoperative continuous monitoring included 5-lead electrocardiography with ST analysis, heart rate, invasive blood pressure, pulse oximetry, body temperature, urinary output, central venous pressure, and pulmonary artery pressures. The depth of anesthesia was measured by a BISTM monitor (Medtronic, Minneapolis, MN, USA).

Regarding vascular access, the patients had arterial lines (radial, femoral, or both in some cases), a central venous catheter (most often through right internal jugular vein), and a Swan–Ganz catheter, as well as 2 peripheral wide-bore iv cannulas. Cardiac output was measured using the thermodilution method at least 3 times (before sternotomy, after weaning from CPB, and after sternal closure), and hemodynamic data were obtained (SVR, PVR, SV, LVSW, and RVSW).

Transesophageal echocardiography was used to assess ventricular and valvular function.

If used, the cardiopulmonary bypass was nonpulsatile, with a flow ranging from 2 to 2.4 L/min/m^2^.

Fluids were administered to ensure hemodynamic stability. Decisions regarding fluid management were based on hemodynamic status, TEE findings describing contractility, and volume status.

The use of inotropic support was based on clinical, laboratory (central venous saturation), and/or TEE criteria of low-cardiac-output syndrome. The inotropes used were dobutamine 3–7 mcg/kg/min, milrinone 0.375–0.75 mcg/kg/min, and levosimendan 0.1–0.2 mcg/kg/min iv.

### 2.9. Postoperative Procedure

Postoperatively, all the patients were transferred to the cardiac ICU. All patients received 300–400 mg of tramadol and metamizole 5 g as a 24 h infusion. The patients received additional morphine boluses 5–10 mg iv at the discretion of the anesthesiologist in duty.

Postoperative low-cardiac-output syndrome (LCOS) was defined as CI < 2.2 L/min/m^2^, pulmonary wedge pressure > 18 mmHg, and mixed venous saturation < 60%. Diagnosis of LCOS was performed after excluding or correcting temperature anomalies (hypothermia), hypovolemia, cardiac rhythm abnormalities, cardiac tamponade, or myocardial ischemia.

By evaluating the total amount of drainage in 12 h, moderate bleeding was defined as 801–1000 mL/12 h, severe bleeding was defined as 1001–2000 mL/12 h, and massive bleeding was defined as >2000 mL/12 h.

Patients were extubated when they met the following predefined criteria: end-tidal carbon dioxide level of <50 mmHg, peripheral oxygen saturation of >90%, fraction of inspired oxygen of <0.4, patient ability to lift head off pillow, patient ability to protect his/her airway, ability to follow commands, no active bleeding (postoperative drainage below 100 mL/h and less than 500 mL over the previous 4 h), hemodynamic stability with or without mild or moderate inotropic support, potassium levels within normal limits, and no metabolic or respiratory acidosis. Moreover, the patients should understand comments and be without postoperative delirium.

### 2.10. Sample Size and Power Analysis

Studies exploring the effects of antioxidants on CS-AKI are rare. One human study used vitamin C before and during cardiac surgery to reduce the incidence of CS-AKI [34]. MitoQ (mitochondria targeted antioxidant, derivate of ubiquinone) showed promising results in an animal model of ischemia–reperfusion injury [19]. Acute kidney injury after surgery varied greatly depending on multiple factors. We assumed that the incidence of CS-AKI in our hospital was approximately 40% and that the intervention group would have an incidence of 15%. We calculated the sample size accordingly. A total of 98 patients should be included, with 49 in each cohort, a study power of 80%, and an alpha error of 0.05.

### 2.11. Statistical Analysis

All data were analyzed using descriptive statistics (R software, version 4.1.0., May 2021. Vienna, Austria, https://www.R-project.org). Continuous variables underwent normality testing using the Shapiro–Wilk test. Normal data were presented as the means and standard deviations. Data without normal distributions were presented as median and inter-quartile range values. The categorical variables were presented as percentages. A *t*-test was used to test the differences in the means between groups and Wilcoxon test values for differences in medians between groups. The independence of categorical data in contingency tables was assessed using Fisher’s exact test or the chi-square test. *p* values below 0.05 were considered statistically significant. Cramér’s V with the confidence interval was used for the effect size of the contingency tables. The minimum value of Cramér’s V was 0, indicating statistical independence. A Cramér’s V value in the range of 0–0.3 indicated a weak effect size, a value from 0.3 to 0.7 signified a medium effect size, and a value greater than 0.7 represented a strong effect size.

## 3. Results

We analyzed data from 73 patients, with 34 patients in the ubiquinone (UQ) group and 39 patients in the placebo group. The groups were well balanced, except for the more frequent anemia among patients in the UQ group. One patient reported gastrointestinal upset during ubiquinone therapy. No other side effects were noted. The general preoperative characteristics of patients are presented in Table 2. Types of surgery and medications are presented in Table 3. None of the patients died during the study. 

### 3.1. Coenzyme Q10 Plasma Levels After One Week of Ubiquinone Therapy

Coenzyme Q10 plasma concentrations were significantly lower in the placebo group compared to patients receiving ubiquinone, as shown in Table 4. Coenzyme Q10 plasma levels in the ubiquinone arm were between 2.719 mg/mL and 57.28 mg/mL, showing a significant variability in absorption.

We measured blood concentrations of CoQ10 after a 24-h period in the cardiac ICU. All patients showed a significant decrease in CoQ10 levels after surgery, with a median CoQ10 concentration (IQR) in the treatment group of 3.694 mg/mL (1.855–5.738). In contrast, the placebo group had a significantly lower concentration (*p* < 0.0001), with a median (IQR) of 0.076 mg/mL (0.00–0.4215). The third measurement was performed in six patients from the treatment group to determine if CoQ10 levels would begin to increase without further therapy. The data are reported in Table 5.

### 3.2. Primary Outcome

#### Incidence of CS-AKI Between Groups

Out of 73 patients in the study, 39.72% of them (N = 29) developed CS-AKI. In the ubiquinone group, 35.29% developed CS-AKI, and 43.58% developed it in the placebo group (*X*^2^(1,N = 73) = 0.4931, *p* = 0.4825); the data are shown in Table 6. The Cramér’s V effect size was small (0.08, CI 95% 0.0–0.313).

### 3.3. Secondary Outcomes

#### 3.3.1. Incidence of AKI Between Groups According to KDIGO Stages

Table 7 shows the distribution of patients according to the KDIGO classification stages of kidney injury. The Fisher’s Exact test result was not significant (*p* = 0.3863).

#### 3.3.2. Blood Loss in the Cardiac ICU

**Figure 2:**
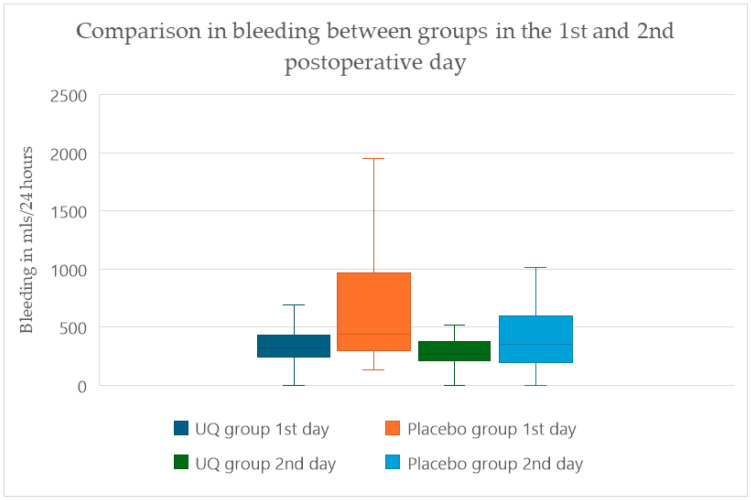
Total postoperative bleeding after 24 and 48 h from mediastinal and thoracic drains. The difference in postoperative bleeding remained statistically significant on the second day after surgery (*p* = 0.0331).

During the first 24 h in the cardiac ICU, patients that did not receive ubiquinone demonstrated a significantly greater blood loss from the thoracic and mediastinal drains, with a median (IQR) drainage of 420 mL (242.5–747.5). Patients receiving ubiquinone therapy showed significantly less bleeding, with a median (IQR) blood loss of 320 mL (230–415; *p* = 0.047). The effect continued in the second postoperative day, as shown in Figure 2. There was no residual effect of heparin after CPB, as evident by normal coagulation parameters after surgery. Figure 2 shows variability between groups.

#### 3.3.3. Hs-Troponin I 24 H After Surgery

**Figure 3:**
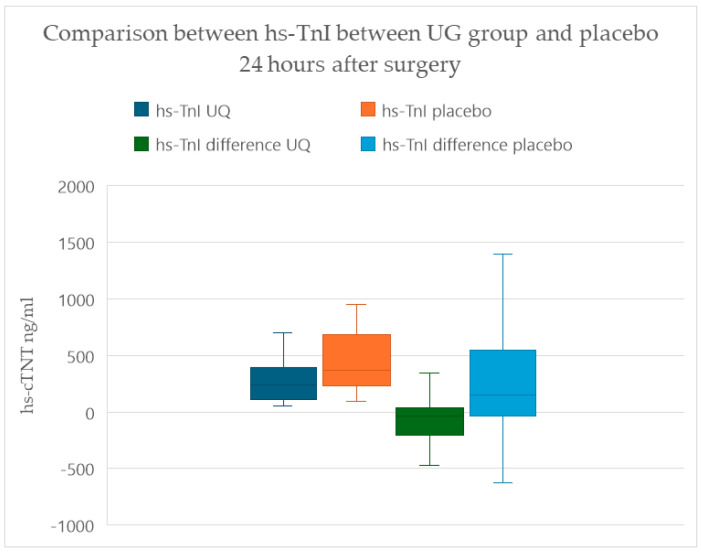
Comparison of postoperative levels of high-sensitivity troponin between groups. Patients receiving ubiquinone showed a significantly lower hs-TnI compared to the placebo group (*p* = 0.024). The differences between preoperative and postoperative hs-TnI in the ubiquinone group showed statistically significant trends toward decreases, while the same differences were not observed in the placebo group (*p* = 0.003).

The median hs-TnI level (IQR) was 239.5 ng/mL (113.25–382.75), which was significantly lower in the UQ group, with a notable postoperative decrease in hs-TnI, as illustrated in Figure 3. Patients administered placebo exhibited a significantly elevated hs-TnI after surgery, with a median (IQR) of 366 (234.5–672.5) ng/mL (*p* = 0.024).

#### 3.3.4. Hemodynamic Parameters After Cardiac Surgery

All patients had an arterial catheter and pulmonary artery catheter in place for invasive hemodynamics during and after the surgery. We compared the mean arterial pressure (MAP), stroke volume index (SVI), cardiac index (CI), systemic vascular resistance (SVR), and pulmonary capillary wedge pressure (PCWP) between groups; the data are presented in Table 8. The stroke volume index was significantly higher in the UQ group.

#### 3.3.5. Preoperative Anemia, Length of Stay (LOS), and Laboratory Data After Surgery

The incidence of anemia was higher in the ubiquinone group at 82.5% compared to the incidence of 51.28% in the placebo group. There was no difference in postoperative anemia between groups, as all the losses were appropriately corrected with blood transfusions. When analyzing our data, 8 out of 12 (66.7%) patients with CS-AKI in the UQ arm were anemic while in the placebo group, and 9 out of 17 (52.94%) patients with CS-AKI after surgery were anemic. This difference was insignificant (*p* = 0.4598). No significant differences were observed in the ICU length of stay and laboratory parameters between groups postoperatively, as indicated in Table 9.

## 4. Discussion

This study investigated the effect of peroral ubiquinone supplementation on cardiac surgery-associated acute kidney injury. While other studies primarily investigated the effects of coenzyme Q10 on heart mitochondrial respiration and cardiac contractility [32], prevention of low cardiac output [35], or prevention of ventricular arrhythmia [36], there was no research examining whether ubiquinone impacted kidney function after cardiac surgery in humans. Schetz. et al. suggested that ubiquinone could be a “missing link” in the prevention of CS-AKI [37]. High-dose ubiquinone supplementation before cardiac surgery was an untested therapy for acute kidney injury prevention. Animal research showed that pretreatment with ubiquinol, a reduced form of ubiquinone, and MitoQ, a ubiquinone derivative, offered protection against kidney ischemia [19,38]. Vitamins E and C showed significant effects in reducing the incidence of contrast-induced acute kidney injury [17]. General preventive strategies were maintaining renal perfusion, avoiding venous congestion, limiting CPB, modifying preoperative anemia, and avoiding nephrotoxic medications [39].

Since patients requiring cardiac surgery often present multiple comorbidities associated with low CoQ10 levels, and since cardiac surgery alone represents significant oxidative stress, we decided to start one-week high-dose ubiquinone therapy.

Patients receiving high doses of ubiquinone showed a trend toward lower incidence of CS-AKI, although this effect was not statistically significant, and Cramér’s V showed a small effect size. Ubiquinone could not prevent a slight increase in creatinine levels postoperatively, but it could provide protection against more significant renal impairment. The possible mechanism was that ubiquinone could provide brief protection against necrosis of renal tubules; however, it did not avert transient tubular dysfunction. Previous studies showed significant improvement in eGFR determined by CKD-EPI in patients receiving ubiquinone and selenium therapy [40]. The limitations of creatinine as a marker for CS-AKI were partially mitigated, as there were no significant differences in body mass index between groups. Additionally, we did not utilize any medications that inhibited the tubular secretion of creatinine.

We compared hemodynamic parameters between groups, as prior studies indicated an increase in ejection fraction and a modest rise in cardiac output [41]. The cardiac index in the UQ group was moderately elevated; however, it did not achieve statistical significance. The postoperative stroke volume index was significantly higher in the UQ group, which was consistent with findings from previous studies [41,42]. This could be explained by the protective effect of UQ during ischemia, the increased ATP production [43], the reduction in lipid peroxidation and oxidative stress [41,44], and possibly the reduction in mitochondrial permeability transition pore necrosis [45,46,47].

Less postoperative bleeding in the UQ group could provide an explanation through ubiquinone’s anti-inflammatory and antioxidant action that supported endothelial function [48]. Reduced bleeding was in concordance with previous work from Makhija et al., but they used a much lower dose of CoQ10 during the same one-week period [36]. Another mechanism of lower postoperative bleeding in the UQ group lied in the effect that ubiquinone had on platelets. Ubiquinone supplementation increased the number of platelets and supported normal ATP production in platelet mitochondria necessary for activation and platelet adhesion, especially under stress [49,50,51].

During coronary artery bypass surgery, myocardial damage is often caused by ischemia–reperfusion injury, hypoperfusion, surgical stress, and the production of reactive oxygen species [52]. A marker of these events was elevated hs-TnI after surgery [53]. The mechanism of ubiquinone myocardial protection could involve the reduction in ischemia–reperfusion injury, oxidative damage, lipid peroxidation, and mitochondrial dysfunction through its antioxidant and anti-inflammatory properties [54]. We found that patients who received ubiquinone therapy had significantly lower levels of hs-TnI after surgery. This was comparable to a study that administered 400 mg of CoQ10 three days prior to surgery [55].

In the current study, we showed that ubiquinone was safe in higher dose ranges and effective in achieving high CoQ10 blood concentrations, despite the complex absorption involving active and passive mechanisms [56]. Due to variable GI absorption, CoQ10 blood concentrations varied significantly between patients in the UQ group. Patients received up to 1000 mg of ubiquinone per day, which was enough to achieve a very high plasma level of CoQ10. Additionally, our data indicated that there was a “procedural dip” or fall in CoQ10 blood concentration 24 h after surgery. This decrease in CoQ10 plasma concentration could be explained by two mechanisms: consumption of antioxidants during and after surgery and redistribution during surgery. Two days after surgery, coenzyme CoQ10 plasma concentration spontaneously recovered, reaching approximately 30–50% of the maximum level observed prior to the procedure. Some patients exhibited a more pronounced drop in CoQ10 blood concentrations. It is to be determined whether the magnitude of this decrease could serve as a predictor for certain postoperative complications.

We identified a potentially unfavorable bias for the patients in the intervention group: a more prevalent anemia. Anemia is a well-known risk factor for acute kidney injury after cardiac surgery and shows significant correlation with postoperative AKI [57]. Red blood cell transfusion is known to independently increase the risk of AKI, which significantly increases in those that develop acute transfusion reactions [58]. The pathophysiology that contributes to the higher susceptibility of anemic patients to develop AKI lies in the reduced oxygen delivery from the generally reduced physiological reserve in these patients and hemodynamic instability [42,59].

The study presented several limitations. A primary limitation was the inability to achieve the calculated sample size owing to patient attrition during follow-up. This resulted in a significantly underpowered analysis of the primary outcome. Five patients withdrew from the study after they received ubiquinone or placebo. Their concerns stemmed from the possibility that the one-week therapy could postpone the surgical procedure. Multiple patients had their surgeries rescheduled to an earlier date for non-medical reasons. One female patient receiving ubiquinone reported gastrointestinal upset that resolved soon after discontinuing therapy. Seven patients from both groups did not adhere to the prescribed therapy. The second limitation was the ability of creatinine as a relatively insensitive marker to detect tubular dysfunction/damage. To detect an increase in serum creatinine, glomerular function had to decrease by as much as 50%, and the increase in serum creatinine was non-linear [60]. Using multiple available biomarkers for tubular cell injury such as Neutrophil Gelatinase-Associated Lipocalin (NGAL) [61], Kidney Injury Molecule-1 (KIM-1) [62], or markers of tubular cell dysfunction, such as α1-microglobulin or β2-microglobulin, would confer more valuable data than the creatinine alone [63]. These were not available for all our patients, so we could not use them. The third limitation was the significant variability in the observed coenzyme Q10 plasma levels following therapy, suggesting that not all patients received the same level of antioxidant protection, which resulted in heterogeneity in the intervention group.

In conclusion, ubiquinone significantly reduced common complications after cardiac surgery, specifically, postoperative bleeding and myocardium damage. It also showed a positive effect on the cardiac hemodynamic parameters. Considering the complex pathophysiology of CS-AKI, ubiquinone exhibited a small effect, which was insufficient to substantially reduce the incidence of CS-AKI in the ubiquinone group. Further research is necessary to determine the impact of ubiquinone on kidney function during cardiac surgery, particularly regarding renal tubule damage and dysfunction.

## Figures and Tables

**Table 1:** Stages of acute kidney injury (KDIGO classification).

Stage	Serum Creatinine	Urine Output
1	1.5–1.9 times baseline OR ≥ 26.5 µmol/L increase	<0.5 mL/kg/h for 6–12 h
2	2.0–2.9 times baseline	<0.5 mL/kg/h for ≥12 h
3	3.0 times baseline OR increase in serum creatinine to ≥353.6 µmol/L OR initiation of renal replacement therapy OR in patients < 18 years, decrease in eGFR to <35 mL/min per 1.73 m^2^	Anuria for 12 h

**Table 2:** General characteristics of patients before surgery.

	UQ	Placebo	*p*-Value
Gender (%)	24 M (70.58)	32 M (82.26)	0.2787
	10 F (29.42)	7 F (17.74)	
Age (years) mean ± SD	67.35 ± 7.68	65.03 ± 8.10	0.2140
BMI	27.82 ± 4.21	28.59 ± 3.99	0.4281
Atrial fibrillation n (%)	3 (8.8)	3 (7.6)	1.000
Arterial hypertension n (%)	31 (91.76)	31 (79.48)	0.2025
Anemia n (%)	28 (82.35)	20 (51.28)	0.0067
Diabetes ID n (%)	9 (25)	9 (23.07)	0.7741
Diabetes II n (%)	11 (32.35)	14 (35.89)	1.000
Previous MI n (%)	7 (25.92)	12 (26.67)	0.6054
Active smoker n (%)	5 (14.7)	11 (28.20)	0.2566
Alcohol n (%)	5 (14.7)	8 (20.51)	0.5568
Hemoglobin g/L mean ± SD.	117.5 ± 14.53	130.05 ± 16.44	0.001
Creatinine µmol/L mean ± SD	84.12 ± 19.27	81.18 ± 12.94	0.4419
Hs TnI (ng/mL) mean ± SD	371.87 ± 424.9	367 ± 548.79	0.9653
Blood glucose (mmol/L) mean ± SD	8.7 ± 2.157	7.7 ± 2.1	0.0521
Ejection fraction %	56.24 ± 10.14	53.74 ±13.87	0.3941
Euroscore II	3.22 ± 1.78	2.44 ± 1.57	0.073

**Table 3:** Types of surgery and medications of interest.

	UQ	Placebo	*p*-Value
Beta blocker n (%)	26 (76.47)	32 (82.05)	0.5764
ACE inhibitor on admission n (%)	17 (50)	22 (56)	0.6425
Statins on admission n (%)	24 (70.5)	33 (84.61)	0.1682
CABG n (%)	22 (64.7)	23 (58.97)	0.8477
VALV n (%)	7 (20.58)	10 (25.64)	
CABG + VALV n (%)	5 (14.7)	5 (12.82)	

**Table 4:** Coenzyme Q10 plasma concentrations after one week of therapy (ubiquinone vs. placebo).

	Ubiquinone Group	Placebo Group	*p*-Value
Coenzyme Q10 plasma levels mg/L	13.76 ± 13.92	0.807 ± 0.72	0.0001

**Table 5:** Plasma CoQ10 concentrations measured at 3 time points. Before surgery, 60 min after admission in the cardiac ICU and 24 h after surgery.

CoQ10 Plasma Concentration (mg/L)			
	Before Surgery	24 h After Surgey	48 h After Surgery
Patient A	57.28	15.71	43.41
Patient B	32.72	6.92	7.58
Patient C	16.15	3.69	4.34
Patient D	14.13	3.45	8.23
Patient E	9.17	0.88	6.67
Patient F	17.98	2.31	6.76

**Table 6:** Incidence of acute kidney injury after surgery AKI; acute kidney injury.

	Ubiquinone	Placebo	*p*-Value
No AKI	N = 22 (64.7%)	N = 22 (56.4%)	0.4825
AKI	N = 12 (35.3%)	N = 17 (43.6%)	

**Table 7:** Differences among groups in the stages by KDIGO classification.

KDIGO Stage	Ubiquinone	Placebo
0	N = 22 (64.7%)	N = 21 (53.8%)
1	N = 11 (32.3%)	N = 12 (30.8%)
2	N = 1 (2.9%)	N = 4 (10.2%)
3	0	N = 2 (5.2%)

**Table 8:** Postoperative hemodynamic parameters and the use of inotropes; MAP, mean arterial pressure; CI, cardiac index; SVI, stroke volume index; SVR, systemic vascular resistance; and PCWP, pulmonary capillary wedge pressure.

Parameter	UQ Group	Placebo Group	*p*-Value
MAP (mmHg)	78.55 ± 8.42	82.18 ± 12.38	0.2102
CI (L/min/m^2^)	3.2320 ± 1.12	2.7204 ± 0.6989	0.0589
SVI (mL/m^2^)	44.26 ± 14.06	34.14 ± 11.87	0.0112
SVR (Dyn*s/cm^5^)	820 ± 329.48	968.42 ± 356.06	0.1367
PCWP (mmHg)	17.90 ± 4.37	17.27 ± 5.45	0.6405
Inotropes N (%)	7 (21.21%)	13 (33.33%)	0.2232

**Table 9:** ICU length of stay (LOS) in hours between groups and postoperative laboratory data.

Parameter	UQ Group	Placebo Group	*p*-Value
ICU LOS	58.09 ± 21.09	60.28 ± 49.41	0.8019
C-reactive protein	195.2 ± 72.83	217.0 ± 94.0	0.3087
Hemoglobin g/L	106.78 ± 9.04	108.22 ± 12.37	0.5697
Platelets count (×10^9^/L)	181.21 ± 80.76	190 ± 56.14	0.5924
Serum creatinine µmol/L	110.88 ± 42.92	107.75 ± 47.66	0.7671
CKD-EPI eGFR (mL/min/m^2^)	73.52 ± 23.58	68.41 ± 23.38	0.3878

## Data Availability

The data presented in this study are available on request from the corresponding author due to ethical, legal, and privacy reasons.
